# Generative AI as an External Cognitive Tool: Cognitive Dependence, AI Literacy, and Perceived Academic Creativity

**DOI:** 10.3390/jintelligence14080185

**Published:** 2026-08-11

**Authors:** Wenzhang Li, Weida Zhang, Mengling Li, Yi Zhang

**Affiliations:** 1 School of Education, Jiangxi Normal University, Nanchang 330022, China; 005848@jxnu.edu.cn; 2 School of Education, Huaibei Normal University, Huaibei 235000, China; 3 Institute of Rural Development, Central China Normal University, Wuhan 430079, China; mengling@mails.ccnu.edu.cn; 4 Department of Human Resources, Jiangxi Normal University, Nanchang 330022, China; jxsdrcb@jxnu.edu.cn

**Keywords:** generative artificial intelligence, cognitive offloading, cognitive dependence, AI literacy, academic creativity, creative cognition, human–AI interaction

## Abstract

Generative artificial intelligence (GenAI) provides a new context for examining how external intelligent systems are associated with human cognitive agency and perceived creative intellectual functioning. Drawing on cognitive offloading theory and AI literacy research, this cross-sectional study tested a moderated mediation model linking GenAI use intensity, GenAI cognitive dependence, AI literacy, and perceived academic creativity. Survey data were collected from 936 undergraduates at six universities in China. Confirmatory factor analysis supported the empirical distinctiveness of the four focal constructs, and latent structural equation modeling was used to examine the hypothesized associations. GenAI use intensity showed a positive direct association with perceived academic creativity and a positive association with GenAI cognitive dependence; cognitive dependence was negatively associated with perceived academic creativity. The standardized direct and indirect associations operated in opposite directions, leaving a small model-implied net association (β ≈ 0.040). AI literacy was negatively associated with cognitive dependence and positively associated with perceived academic creativity. The use-dependence association was weaker at higher levels of AI literacy, and the negative indirect association through dependence was correspondingly smaller. These cross-sectional results are interpreted as associations and do not establish temporal or causal ordering.

## 1. Introduction

Generative artificial intelligence (GenAI) has created a new setting for studying human intelligence in interaction with external cognitive systems. Large language models can generate text, explain concepts, propose arguments, summarize information, revise writing, translate content, produce code, and simulate dialog (Chan & Hu, 2023; Kasneci et al., 2023; Tlili et al., 2023; H. Wang et al., 2024). These systems do more than provide access to information. They participate in the cognitive work through which students search, organize, evaluate, and produce academic ideas. GenAI therefore offers a timely case for examining how intelligent tools may extend, redirect, or displace human cognitive activity.

The relevance of this issue extends beyond educational technology. Intelligence research has long been concerned with how people solve problems, use tools, regulate cognition, and adapt to changing environments. GenAI intensifies these questions because it can support higher-order processes that were previously more clearly located within the learner, such as generating hypotheses, integrating evidence, evaluating arguments, and producing creative alternatives. In this study, we use higher education as an applied context for examining the relation between GenAI use, cognitive dependence, AI literacy, and perceived academic creativity.

GenAI may support creative intellectual functioning by lowering the entry barrier to complex tasks, providing examples, stimulating ideation, offering feedback, and helping students compare alternative explanations (Farrokhnia et al., 2024; Kasneci et al., 2023; Su & Yang, 2023). Recent work also indicates that large language models can support self-regulated learning and classroom dialog analysis when embedded in meaningful learning designs (Lan & Zhou, 2025; Y. Long et al., 2024). In this sense, GenAI can operate as an external cognitive scaffold that expands the resources available for academic thinking.

At the same time, GenAI may weaken the internal cognitive engagement required for durable learning and creative development. Perceived academic creativity refers here to students’ self-reported capacity to generate novel and useful academic ideas, such as proposing original questions, integrating knowledge across fields, identifying limitations in arguments, constructing new explanations, and developing innovative approaches to research or problem solving (Karunarathne & Calma, 2024; Kaufman, 2012). This construct is treated as a self-reported indicator of creative intellectual functioning in academic contexts rather than as an objective measure of creative intelligence. It is important because academic creativity requires not only polished outputs but also problem finding, epistemic struggle, evaluative judgment, and intellectual ownership.

Accordingly, the outcome captures students’ creative self-beliefs and perceived competence in academic work. Such perceptions may overlap with performance-based creativity, but they should not be treated as a direct measure of objective creative capacity.

Evidence on AI and creativity remains mixed. GenAI systems can perform well on divergent-thinking tasks and may improve the apparent fluency, quality, or originality of creative products (Arora et al., 2025; Hubert et al., 2024; Koivisto & Grassini, 2023; D. Wang et al., 2026). Doshi and Hauser (2024), for example, found that AI-generated ideas improved individual creative writing, particularly among less creative writers, while also reducing the collective diversity of outputs. This tension suggests that AI-assisted product quality and students’ own perceived creative functioning should be examined separately.

A central distinction in this study is between GenAI use intensity and GenAI cognitive dependence. GenAI use intensity refers to how frequently and broadly students use GenAI for learning-related purposes, such as understanding course content, summarizing information, generating outlines, revising writing, and solving academic problems. GenAI cognitive dependence refers to students’ tendency to outsource core cognitive processes to GenAI, including idea generation, information integration, logical organization, judgment verification, and confidence regulation. Frequent use may indicate active engagement with a powerful external tool; cognitive dependence indicates a possible shift in cognitive agency from the learner to the system. We define scaffolded use as assistance that extends students’ thinking while they retain control over goals, evidence, evaluation, and authorship. Substitutive use occurs when GenAI performs the core cognitive work that the learner would otherwise undertake.

This distinction is grounded in cognitive offloading theory. Cognitive offloading refers to the use of external tools to reduce internal cognitive effort (Risko & Gilbert, 2016). Offloading can be adaptive when it extends human capability, as in the use of notes, diagrams, calculators, databases, and search engines. However, when offloading replaces rather than supports internal processing, it may be associated with weaker memory, metacognitive monitoring, conceptual understanding, and independent problem solving (Sparrow et al., 2011; Ward, 2021). GenAI extends this concern because it can generate answers, organize arguments, simulate reasoning, and provide persuasive explanations. Students may therefore offload not only information retrieval but also the higher-order processes through which creativity develops.

Recent findings support the need to examine this dependence pathway. Fan et al. (2025) introduced metacognitive laziness in GenAI-supported learning and found that AI assistance may be linked to dependence on external support and weaker deep engagement. Bastani et al. (2025) reported that generative AI without appropriate guardrails improved practice performance but was followed by poorer performance when AI support was removed. These findings point to a performance illusion: students may appear more capable while using AI, even when the cognitive processes needed for independent performance remain underdeveloped.

AI literacy may help explain why similar levels of GenAI use are associated with different cognitive patterns. AI literacy generally refers to the knowledge, skills, and dispositions needed to understand, evaluate, use, and critically engage with AI systems (D. Long & Magerko, 2020; Ng et al., 2021). In GenAI contexts, AI literacy includes identifying learning needs, formulating prompts, evaluating generated content, recognizing hallucinations and bias, understanding academic integrity boundaries, and retaining responsibility for one’s own reasoning (Jin et al., 2025; Laupichler et al., 2023; H. Wang et al., 2024). Thus, AI literacy is not only a technical competence but also a cognitive-regulatory and epistemic capacity.

Although research on GenAI in education has expanded rapidly, several gaps remain for intelligence and learning research. First, many studies focus on acceptance, perceived usefulness, academic integrity, or performance outcomes, with less attention to psychological mechanisms linking GenAI use to students’ perceived creative intellectual functioning (Chan & Hu, 2023; Strzelecki, 2024; Tlili et al., 2023). Second, AI overreliance is often discussed broadly, but behavioral use intensity is rarely separated from cognitive dependence. Third, AI literacy is widely promoted, yet more empirical evidence is needed on whether it moderates the pathway from GenAI use to dependence. Fourth, much work on AI and creativity asks whether AI systems can be creative; less is known about whether students continue to experience themselves as capable creative thinkers while using AI.

The present study addresses these gaps by testing a moderated mediation model among university students. We examine whether GenAI use intensity is indirectly associated with perceived academic creativity through GenAI cognitive dependence and whether AI literacy moderates the association between GenAI use intensity and cognitive dependence. The model captures competing pathways: GenAI use intensity may be positively associated with perceived academic creativity when it provides ideational support, examples, explanations, and feedback; it may also show a negative indirect association when students rely on AI to replace core academic thinking processes.

**Figure 1:**
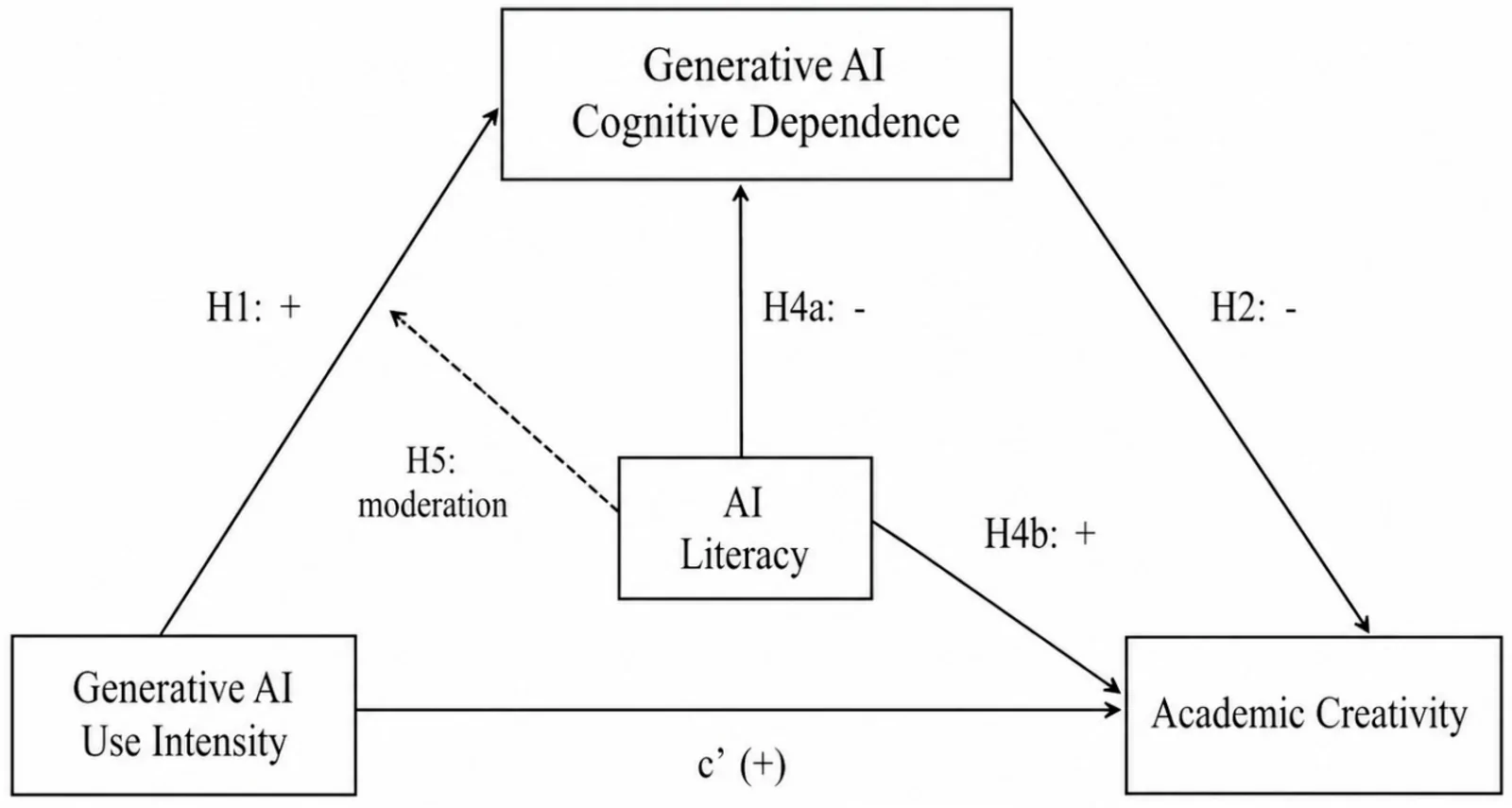
Hypothesized moderated mediation model. Note. The model is interpreted as a theoretically informed cross-sectional association model. GAIU = GenAI use intensity; GCD = GenAI cognitive dependence; AIL = AI literacy; PAC = perceived academic creativity. H3 denotes the indirect association; H6 denotes the conditional indirect association.

This study contributes to the literature in three ways. First, it introduces GenAI cognitive dependence as a mechanism through which an external intelligent system may be linked to weaker perceived creative intellectual functioning. Second, it distinguishes GenAI use intensity from GenAI cognitive dependence, clarifying the difference between using AI as a cognitive scaffold and relying on AI as a substitute for independent thought. Third, it examines AI literacy as a moderator of the dependence pathway, providing evidence that the consequences of GenAI use depend on students’ capacity to regulate the boundary between external assistance and internal cognitive agency. Accordingly, the hypothesized moderated mediation model linking GenAI use intensity, GenAI cognitive dependence, AI literacy, and perceived academic creativity is presented in Figure 1.

### Hypotheses

**H1.**  *GenAI use intensity is positively associated with GenAI cognitive dependence.*

**H2.**  *GenAI cognitive dependence is negatively associated with perceived academic creativity.*

**H3.**  *GenAI use intensity has a negative indirect association with perceived academic creativity through GenAI cognitive dependence.*

**H4a.**  *AI literacy is negatively associated with GenAI cognitive dependence.*

**H4b.**  *AI literacy is positively associated with perceived academic creativity.*

**H5.**  *AI literacy moderates the association between GenAI use intensity and GenAI cognitive dependence, such that the positive association is weaker among students with higher AI literacy.*

**H6.**  *The negative indirect association between GenAI use intensity and perceived academic creativity through cognitive dependence is weaker at higher levels of AI literacy.*

## 2. Method

### 2.1. Participants and Procedure

This study used a cross-sectional survey design to examine associations among GenAI use intensity, GenAI cognitive dependence, AI literacy, and perceived academic creativity. The design does not establish temporal ordering among the constructs; consequently, the hypothesized directional paths are treated as theoretically informed statistical associations. Participants were recruited from six universities in China using stratified convenience sampling. To increase sample heterogeneity, recruitment covered different academic years and disciplinary backgrounds, including humanities and social sciences, education and psychology, science and engineering, economics and management, and other fields.

The initial sample consisted of 1024 undergraduate students. Before analysis, responses were screened for data quality. Participants were excluded if they failed either of two attention-check items, completed the questionnaire in an unrealistically short time, selected the same response option for nearly all scale items, or had excessive missing data. After data screening, the final analytic sample consisted of 936 valid responses.

Participants ranged in age from 18 to 25 years (M = 20.42, SD = 1.38). Among them, 408 were male (43.6%), 520 were female (55.6%), and 8 selected other or prefer not to say (0.8%). In terms of academic year, 247 were freshmen (26.4%), 276 were sophomores (29.5%), 238 were juniors (25.4%), and 175 were seniors or above (18.7%). Regarding disciplinary background, 31.2% majored in humanities and social sciences, 18.6% in education or psychology, 29.8% in science and engineering, 12.7% in economics or management, and 7.7% in other disciplines. All participants reported having used at least one GenAI tool, such as ChatGPT, DeepSeek, Kimi, Wenxin Yiyan, Tongyi Qianwen, Claude, or Gemini, for learning-related purposes during the previous six months.

The survey was administered through a secure online questionnaire platform. Before completing the questionnaire, participants read an informed consent statement explaining the study purpose, voluntary participation, anonymity, confidentiality, and the right to withdraw at any time. No personally identifiable information was collected. To reduce common method bias, the questionnaire was divided into separate sections, the order of constructs was arranged to reduce demand characteristics, and participants were informed that there were no right or wrong answers. The study protocol was approved by the Ethics Committee of Jiangxi Normal University on 1 August 2025.

### 2.2. Measures

Unless otherwise specified, all items were rated on a 5-point Likert scale ranging from 1 = strongly disagree to 5 = strongly agree. Higher scores indicated higher levels of the corresponding construct. Because several measures were adapted or contextualized for GenAI-supported learning, scale-score interpretation is specific to the current higher education context. The full item wording and adaptation notes are provided in Supplementary Materials.

#### 2.2.1. GenAI Use Intensity

GenAI use intensity was measured with six items developed for the present study based on prior research on students’ GenAI use, technology-supported academic writing, and learning support in higher education (Chan & Hu, 2023; Kim et al., 2025; Strzelecki, 2024). The items assessed the frequency and breadth of students’ use of GenAI tools for learning-related tasks, including understanding course content, completing assignments, summarizing information, generating outlines, revising written work, and solving complex academic problems. Sample items included “I use generative AI tools to help me complete assignments, reports, or course papers” and “When a learning task is complex, I usually use generative AI to obtain assistance.” Items were rated from 1 = never to 5 = always. In the structural equation model, GenAI use intensity was specified as a latent variable with six observed indicators. Because this construct already includes frequency-related content, weekly GenAI use frequency was not entered as a separate control variable in the main model to avoid conceptual overlap and overcontrol.

#### 2.2.2. AI Literacy

AI literacy was assessed using a 16-item scale adapted from AI literacy and GenAI literacy frameworks (Jin et al., 2025; Laupichler et al., 2023; D. Long & Magerko, 2020; Ng et al., 2021). The scale measured students’ capacity to use GenAI critically, effectively, and responsibly for learning purposes. Four content domains were represented: needs analysis, prompt and language skills, autonomous learning, and critical evaluation. Needs analysis refers to students’ ability to identify learning needs before using GenAI. Prompt and language skills refer to their ability to formulate, refine, and adjust prompts. Autonomous learning refers to the ability to integrate GenAI into self-directed learning without losing learning agency. Critical evaluation refers to the ability to judge the accuracy, limitations, and academic appropriateness of AI-generated content. Sample items included “Before using generative AI, I can identify what learning problem I need to solve,” “I can adjust my prompts to make generative AI responses better fit the learning task,” and “I critically evaluate the accuracy of generative AI-generated content.” In the main structural equation model (SEM), AI literacy was represented as an overall latent construct because the hypotheses concerned students’ general regulatory capacity in GenAI-supported learning. The domain structure and construct-level representation are summarized in Supplementary Table S2.

#### 2.2.3. GenAI Cognitive Dependence

GenAI cognitive dependence was measured with a 12-item scale adapted from research on cognitive offloading, technology dependence, and AI-supported learning (Risko & Gilbert, 2016; Sparrow et al., 2011; Ward, 2021). The construct was defined as students’ tendency to outsource core cognitive processes to GenAI, including idea generation, information integration, logical organization, and judgment verification. The content domains included cognitive outsourcing dependence, uncritical acceptance of AI outputs, and weakened independent thinking. Sample items included “When I encounter a learning problem, my first reaction is usually to ask generative AI rather than think it through myself,” “I often rely on generative AI to generate the basic ideas for assignments, papers, or reports,” and “Without generative AI, I feel less confident in completing complex learning tasks.” Higher scores indicated stronger cognitive dependence on GenAI. The domain structure and construct-level representation are summarized in Supplementary Table S2.

#### 2.2.4. Perceived Academic Creativity

Perceived academic creativity was measured using 11 items adapted from the scholarly creativity domain of the Kaufman Domains of Creativity Scale and revised to fit the higher education learning context (Kaufman, 2012). The scale assessed students’ self-reported capacity to generate novel and useful academic ideas in course learning, academic writing, and research-related tasks. Sample items included “I can propose original questions in course learning,” “I can connect knowledge from different courses or disciplines to form new ideas,” and “I can identify limitations in existing arguments, materials, or studies.” Higher scores indicated higher perceived academic creativity. The construct is interpreted as self-reported creative intellectual functioning in academic contexts rather than as objectively assessed creative performance or a direct measure of general intelligence.

#### 2.2.5. Control Variables

Several demographic and learning-related variables were included as controls: gender, academic year, disciplinary background, self-reported academic performance, length of GenAI use, and prior participation in AI-related training. Gender was represented by indicators for female and other/prefer not to say, with male as the reference group. Academic year was represented by sophomore, junior, and senior-or-above indicators, with freshman as the reference group. Discipline was represented by education/psychology, science/engineering, economics/management, and other-discipline indicators, with humanities and social sciences as the reference group. Length of GenAI use was dummy-coded with less than one month as the reference group. Weekly GenAI use frequency was excluded from the primary model because it overlapped conceptually and empirically with the use-intensity construct; a supplementary sensitivity model added it as a control.

#### 2.2.6. Scale Adaptation and Content Validation

Because several measures were adapted for GenAI-supported higher education, scale adaptation preceded the formal study. An initial item pool was generated from prior literature on GenAI use, AI literacy, cognitive offloading, technology dependence, and academic creativity. Items were contextualized for learning-related GenAI use and reviewed for conceptual relevance, clarity, and appropriateness in Chinese higher education. A separate pilot sample of 96 undergraduates then completed the draft questionnaire; these cases were not included in the formal sample. The pilot showed no item-level missing responses, scale α coefficients from 0.852 to 0.937, corrected item-total correlations from 0.489 to 0.738, and item means within the interior of the five-point response range. These checks supported item comprehension, response feasibility, and preliminary internal consistency. Formal psychometric evaluation and cross-validation were conducted with the 936-case main sample. Full item wording, construct domains, and adaptation notes are provided in Supplementary Materials, and pilot diagnostics are reported in Supplementary Table S3.

### 2.3. Data Analysis

Data analyses were conducted using SPSS 26.0, Mplus 8.9, and R 4.3.2. Data screening examined missing values, careless responses, outliers, and distributional characteristics. Item-level missingness was low, and full information maximum likelihood estimation was used to handle missing data. Because mild multivariate non-normality was detected, robust maximum likelihood (MLR) estimation was used for CFA and latent SEM.

Descriptive statistics and Pearson correlations were calculated for the main variables. Internal consistency was evaluated using Cronbach’s α, McDonald’s ω, and corrected item-total correlations. Convergent validity was assessed using standardized factor loadings, composite reliability, and average variance extracted. Discriminant validity was evaluated using the heterotrait–monotrait ratio (HTMT), competing measurement models, and an item-level four-factor exploratory factor analysis (EFA) with MLR extraction and Geomin oblique rotation.

Confirmatory factor analysis (CFA) examined the measurement properties of the four focal constructs. To reduce same-sample confirmation concerns, the 936 cases were divided into calibration and validation samples of 468 using seed 20250801 and Python 3.13.5 random.Random (MT19937). The exact case assignment and generation script are retained with the analysis files. The same four-factor MLR CFA was estimated independently in both halves.

Fourth, a series of competing measurement models was tested to examine whether the four constructs were empirically distinguishable. The hypothesized four-factor model was compared with alternative models, including a three-factor model combining GenAI use intensity and GenAI cognitive dependence, a three-factor model combining AI literacy and perceived academic creativity, a two-factor model, and a one-factor model. This step was important because GenAI use intensity and GenAI cognitive dependence are conceptually related but theoretically distinct.

Fifth, common method bias was addressed through both procedural and statistical remedies. Procedurally, participants completed the questionnaire anonymously, scale items were separated into different sections, and participants were informed that there were no right or wrong answers. Statistically, Harman’s single-factor test, a single-factor CFA model, and an unmeasured latent method factor sensitivity check were used to assess the potential influence of common method variance. Because Harman’s test is limited, the single-factor CFA and method-factor sensitivity checks were treated as more informative evidence.

Finally, the hypothesized moderated mediation model was estimated using latent structural equation modeling. The latent interaction between GenAI use intensity and AI literacy was estimated with the latent moderated structural equations (LMS) approach. The interaction model was compared with a genuinely nested model in which the interaction path to cognitive dependence was fixed to zero. Because LMS models do not provide conventional global fit indices, the two MLR models were compared using their H0 loglikelihoods, scaling correction factors, information criteria, and the MLR-scaled likelihood-ratio test. Conditional slopes and conditional indirect associations were used for interpretation. Supplementary sensitivity analyses added weekly GenAI-use frequency to observed-score models with the same controls and examined university-level intraclass correlations.

Because the present study used a cross-sectional survey design, the path and indirect-effect terminology denotes model-implied associations. The analyses cannot establish temporal sequence, causal mediation, or the direction of influence among the constructs.

## 3. Results

### 3.1. Preliminary Analyses

Prior to hypothesis testing, the data were screened for missing values, outliers, distributional characteristics, and response quality. Item-level missingness was below 1.5% and was handled using full information maximum likelihood estimation. Skewness values ranged from −0.58 to 0.64, and kurtosis values ranged from −0.71 to 0.82, suggesting no serious violation of univariate normality. Because mild multivariate non-normality was detected, robust maximum likelihood estimation was used in subsequent latent variable analyses.

Descriptive statistics and correlations among the focal variables are presented in Table 1. GenAI use intensity was positively correlated with GenAI cognitive dependence and AI literacy. GenAI cognitive dependence was negatively correlated with perceived academic creativity, whereas AI literacy was positively correlated with perceived academic creativity and negatively correlated with cognitive dependence. These correlations were consistent with the hypothesized model.

### 3.2. Reliability, Convergent Validity, and Domain Structure

The reliability and convergent validity of the study variables were examined before testing the structural model. As shown in Table 2, all constructs demonstrated satisfactory internal consistency. Cronbach’s alpha values ranged from 0.887 to 0.947, McDonald’s omega values ranged from 0.891 to 0.949, and composite reliability values ranged from 0.889 to 0.948. All standardized factor loadings exceeded 0.60, and AVE values ranged from 0.535 to 0.573, supporting acceptable convergent validity. Corrected item-total correlations ranged from 0.621 to 0.834, suggesting that all items contributed adequately to their corresponding constructs. Standardized factor loading ranges are summarized in Table 2, and full item wording is provided in Supplementary Materials.

Because AI literacy and GenAI cognitive dependence were conceptualized with multiple content domains, their domain structures were used to guide item development and interpretation. In the main SEM, both constructs were represented as overall latent variables because the hypotheses concerned overall AI literacy and overall cognitive dependence. The domain structure and construct-level representation are summarized in Supplementary Table S2.

### 3.3. Measurement Model, Discriminant Validity, and Common Method Bias

A confirmatory factor analysis was conducted to test the hypothesized four-factor measurement model, in which GenAI use intensity, AI literacy, GenAI cognitive dependence, and perceived academic creativity were specified as four distinct latent constructs. The four-factor model demonstrated good fit to the data, χ^2^(939) = 2034.68, *p* < .001, CFI = 0.955, TLI = 0.952, RMSEA = 0.035, and SRMR = 0.041.

To further evaluate discriminant validity, the hypothesized four-factor model was compared with several alternatives. As shown in Table 3, it fit substantially better than models that combined GenAI use intensity with cognitive dependence, combined AI literacy with perceived academic creativity, or collapsed the constructs further. HTMT values ranged from 0.198 to 0.554; the value for the conceptually closest pair, GenAI use intensity and cognitive dependence, was 0.471. All values were below 0.85, supporting discriminant validity.

A reproducible split-sample CFA provided an additional check on measurement stability. CASEIDs were shuffled with Python 3.13.5 random.Random (MT19937), seed 20250801; the first 468 formed the calibration sample and the remaining 468 the validation sample. Both MLR models terminated normally. Calibration fit was χ^2^(939) = 695.573, *p* = 1.000, CFI = 1.000, raw TLI = 1.021, RMSEA = 0.000, 90% CI [0.000, 0.000], and SRMR = 0.024. Validation fit was χ^2^(939) = 695.990, *p* = 1.000, CFI = 1.000, raw TLI = 1.021, RMSEA = 0.000, 90% CI [0.000, 0.000], and SRMR = 0.024. TLI values above 1.00 were bounded at 1.000 for presentation while the raw values were retained in the output. The bounded indices coincide because they reached their conventional upper or lower limits; the unrounded χ^2^ values and loglikelihoods differ across samples. See Supplementary Table S4.

A four-factor EFA of all 45 items (N = 936) used MLR extraction and Geomin oblique rotation. Primary loadings ranged from 0.621 to 0.819, and the largest absolute cross-loading was 0.046 (PAC9 on the GAIU-aligned factor). The complete 45-by-4 loading matrix and factor correlation matrix are reported in Supplementary Table S5; no loading was suppressed. Together with HTMT(GAIU, GCD) = 0.471, these results provide item-level evidence that use intensity and cognitive dependence were empirically distinguishable.

Common method bias was also examined using procedural and statistical approaches, as summarized in Table 4. Harman’s single-factor test indicated that the first unrotated factor accounted for 31.84% of the total variance, below the commonly used 40% threshold. The single-factor CFA model showed poor fit, χ^2^(945) = 10,386.17, CFI = 0.611, TLI = 0.593, RMSEA = 0.103, and SRMR = 0.137. A method-factor sensitivity check further indicated that the direction and statistical significance of the focal structural paths were not substantially altered when a common latent method factor was included. The available evidence therefore suggests that common method variance was unlikely to fully account for the observed associations. Nevertheless, because all focal variables were measured by self-report at one time point, common method bias cannot be ruled out completely and is addressed as a limitation.

### 3.4. Structural Model

The hypothesized structural model was tested after establishing the adequacy of the measurement model. The structural model without the latent interaction term showed good fit to the data, χ^2^(963) = 2258.14, *p* < .001, CFI = 0.950, TLI = 0.947, RMSEA = 0.038, and SRMR = 0.045.

Standardized path coefficients are presented in Table 5. GenAI use intensity was positively associated with GenAI cognitive dependence, β = 0.36, SE = 0.04, *p* < .001, indicating that students who used GenAI more frequently and broadly tended to report stronger cognitive dependence. AI literacy was negatively associated with cognitive dependence, β = −0.40, SE = 0.03, *p* < .001, suggesting that students with higher AI literacy were less likely to report outsourcing core cognitive processes to GenAI. GenAI cognitive dependence was negatively associated with perceived academic creativity, β = −0.39, SE = 0.04, *p* < .001. AI literacy was positively associated with perceived academic creativity, β = 0.42, SE = 0.04, *p* < .001. GenAI use intensity also had a positive direct association with perceived academic creativity after accounting for cognitive dependence and AI literacy, β = 0.18, SE = 0.04, *p* < .001. Based on the reported standardized solution, the model accounted for 30% of the variance in GenAI cognitive dependence and 43% of the variance in perceived academic creativity.

As a sensitivity check, observed-score models using standardized scale means, the same controls, and HC3 standard errors were re-estimated with weekly GenAI-use frequency included. For cognitive dependence, the GAIU coefficient changed from 0.539 to 0.534, the AIL coefficient from −0.467 to −0.468, and the GAIU-by-AIL coefficient from −0.146 to −0.146. For perceived academic creativity, the GAIU, AIL, and GCD coefficients changed from 0.347, 0.211, and −0.519 to 0.346, 0.211, and −0.519. Weekly frequency was not associated with either outcome after these variables were included (β = 0.008, *p* = .826 for GCD; β = 0.002, *p* = .961 for PAC). See Supplementary Table S6.

University-level clustering was also examined. With 156 participants at each of six universities, one-way ICC estimates were 0.003 for GAIU, less than 0.001 for AIL, zero for GCD after the negative sampling estimate was bounded at zero, and less than 0.001 for PAC. These values indicated negligible between-university variance in the focal scale scores, while the small number of universities limits broader institutional inference (Supplementary Table S8).

### 3.5. Mediation and Moderated Mediation Analyses

The indirect association between GenAI use intensity and perceived academic creativity through GenAI cognitive dependence was first examined. The indirect effect was significant, β = −0.140, SE = 0.025, 95% CI [−0.191, −0.094]. This finding indicates that higher GenAI use intensity was associated with stronger cognitive dependence, which in turn was associated with lower perceived academic creativity.

At the same time, the direct association between GenAI use intensity and perceived academic creativity remained positive and significant, β = 0.18, *p* < .001. Based on the standardized direct and indirect estimates, the model-implied net association was small (β ≈ 0.040), because the direct and indirect associations operated in opposite directions. This result is best interpreted as an inconsistent mediation or competing-pathway pattern rather than as a conventional partial mediation model. The finding does not indicate that frequent GenAI use is uniformly beneficial or uniformly detrimental; rather, it suggests that GenAI use may be associated with perceived academic creativity through different psychological pathways. The standardized direct and indirect associations are summarized in Table 6.

Next, the moderating association of AI literacy was examined. In the nested-model specification, the freely estimated latent interaction was negative, b = −0.244, SE = 0.039, *p* < .001 (Mplus STDYX β = −0.155), indicating that the positive use-intensity/dependence association was smaller at higher AI literacy. The restricted model (interaction path fixed to zero) had H0 loglikelihood −46,419.648, scaling factor 0.9503, and 169 free parameters; the free model had H0 loglikelihood −46,401.249, scaling factor 0.9485, and 170 free parameters. The MLR-scaled likelihood-ratio test favored the free model, Δχ^2^(1) = 57.113, *p* < .001. AIC decreased from 93,177.296 to 93,142.497 and BIC from 93,995.529 to 93,965.572. The variance explained in cognitive dependence increased from 0.497 to 0.520 (ΔR^2^ = 0.023), providing an additional magnitude indicator. Full model-comparison details and Mplus outputs are provided in Supplementary Table S7 and the reproducibility files.

Simple slope analyses showed that the association between GenAI use intensity and cognitive dependence was strongest among students with low AI literacy, β = 0.52, SE = 0.05, *p* < .001. The association remained significant but weaker at the mean level of AI literacy, β = 0.34, SE = 0.04, *p* < .001. Among students with high AI literacy, the association was further reduced, β = 0.16, SE = 0.05, *p* = .002. These findings supported the hypothesized moderating role of AI literacy.

Conditional indirect effects were then examined to test the moderated mediation hypothesis. As shown in Table 7, the negative indirect association between GenAI use intensity and perceived academic creativity through cognitive dependence was strongest when AI literacy was low, weaker when AI literacy was at the mean level, and weakest when AI literacy was high. The index of moderated mediation was significant, β = 0.070, SE = 0.018, 95% CI [0.037, 0.108], indicating that the indirect association varied as a function of AI literacy.

## 4. Discussion

The findings support a distinction between the breadth of GenAI use and the cognitive relationship students develop with the tool. The positive direct and negative dependence-related associations largely offset one another, leaving a small net association. This pattern shifts the theoretical focus from how much students use GenAI to whether external assistance remains integrated with their own judgment, authorship, and cognitive agency.

For intelligence and educational psychology, the competing-pathway pattern is more informative than a uniformly positive or negative account. GenAI can be used in scaffolded ways that extend an ongoing reasoning process, or in substitutive ways that transfer core intellectual work to the system. The cross-sectional data do not show that either pattern causes changes in creativity, but they identify associations that can be tested prospectively and experimentally.

### 4.1. GenAI as an External Cognitive Tool and Human Cognitive Agency

The positive direct association between GenAI use intensity and perceived academic creativity is consistent with the view that intelligent tools can extend human cognitive resources. GenAI can help students start difficult tasks, receive immediate feedback, compare explanations, generate alternatives, and revise academic work (Chan & Hu, 2023; Kasneci et al., 2023; Kim et al., 2025; Tlili et al., 2023). These affordances may support creative intellectual functioning when students use AI to broaden the range of ideas they consider and to refine their own academic products.

Yet this positive pathway should not be interpreted as evidence that more AI use automatically produces stronger creativity. The simultaneous positive association between GenAI use intensity and cognitive dependence qualifies the interpretation. GenAI differs from many earlier digital tools because it can generate research questions, propose arguments, synthesize sources, evaluate drafts, and simulate reasoning. These functions make it especially powerful as a scaffold, but they also make it possible for students to outsource the central cognitive work that creative academic development requires.

The findings therefore support a distinction between scaffolded use and substitutive use. Scaffolded use occurs when students use GenAI to extend thinking while retaining control over goals, criteria, evidence, and reasoning. Substitutive use occurs when GenAI performs the core cognitive work that learners are expected to develop. This distinction is directly relevant to intelligence research because it concerns the boundary between external cognitive support and internal cognitive agency. GenAI may change not only task performance, but also students’ perceived ownership of the processes through which they think, judge, and create.

### 4.2. Cognitive Dependence and Perceived Creative Intellectual Functioning

The negative association between GenAI cognitive dependence and perceived academic creativity is theoretically important. Academic creativity involves problem finding, conceptual integration, originality, evaluative judgment, uncertainty tolerance, and defensible academic positions (Karunarathne & Calma, 2024; Kaufman, 2012). Students who reported relying on GenAI for initial ideas, argument structures, claim verification, or confidence also reported lower perceived creative agency. The direction may also run in reverse: students who already perceive themselves as less creative may be more inclined to rely on GenAI for these functions.

This pattern helps reconcile mixed evidence on GenAI and creativity. Some studies show that GenAI can enhance creative outputs by providing suggestions, examples, and linguistic resources (Doshi & Hauser, 2024). Other studies warn that AI-generated assistance may reduce diversity, originality, or sustained effort (Doshi & Hauser, 2024; Yan et al., 2024). The present study extends this debate by shifting attention from AI-generated products to students’ own perceived creative intellectual functioning. For human intelligence research, the key issue is not only whether AI can generate creative ideas, but whether learners continue to develop the capacity to question, evaluate, transform, and author ideas while using AI.

The small model-implied net association is substantively important. A positive direct association and a negative dependence-related association operated in opposite directions, so statistical significance should not be read as evidence of a broadly positive effect. The result instead indicates heterogeneity in the psychological pathways accompanying GenAI use and supports a more restrained interpretation of its relation to perceived academic creativity.

### 4.3. AI Literacy as Cognitive Regulation

The moderating role of AI literacy clarifies why similar levels of GenAI use may correspond to different dependence patterns. Students with higher AI literacy showed a weaker association between GenAI use intensity and cognitive dependence. This finding supports the view that AI literacy is not merely familiarity with tools or competence in prompt engineering. It functions as a cognitive-regulatory and epistemic capacity that helps learners evaluate AI outputs, recognize limitations, verify information, and maintain responsibility for academic reasoning (Jin et al., 2025; D. Long & Magerko, 2020; Ng et al., 2021).

This regulatory interpretation is important because efficiency-oriented AI training may unintentionally make students better at depending on AI. Students may learn to produce better prompts and faster outputs without learning when to pause, reason independently, or reject generated content. The present findings suggest that AI literacy should include cognitive boundary-setting: knowing which parts of a task can be supported by GenAI and which parts should remain under the learner’s own intellectual control. This boundary-setting function is central to preserving creative cognition in human–AI environments.

The moderated mediation result indicates that the negative indirect association through cognitive dependence was smaller at higher AI literacy. This pattern does not establish that AI literacy prevents dependence. It suggests that frequent use and substitutive reliance were less strongly coupled among students reporting greater critical evaluation, verification, and responsibility for academic reasoning.

### 4.4. Implications for Human–AI Learning and Assessment

The findings have implications for designing environments in which external intelligent tools support rather than displace human intellectual development. First, policies and course guidelines should distinguish constructive AI assistance from cognitive substitution. Constructive assistance may include using GenAI to clarify concepts, generate counterarguments, compare explanations, or obtain formative feedback. Cognitive substitution may include allowing GenAI to provide the central idea, determine the argument structure, verify claims without checking, or replace independent judgment.

Second, assessment should focus on the creative process as well as the final product. In GenAI-rich environments, polished outputs alone are no longer sufficient evidence of students’ cognitive engagement. Students may be asked to document preliminary thinking, prompt logs, annotated AI outputs, source-checking records, draft histories, revision memos, and statements of intellectual contribution. The purpose of such evidence is not merely surveillance; it is to make students’ reasoning visible and to ensure that external assistance supports rather than replaces creative academic work.

Third, AI literacy education should be framed as training in cognitive regulation rather than as technical tool instruction alone. Students with lower AI literacy may need explicit support in hallucination detection, source verification, task-boundary setting, academic integrity, and independent thinking routines. Students with higher AI literacy may benefit from more advanced human–AI collaboration tasks, such as using GenAI to generate counterarguments, compare theoretical frameworks, or test alternative research designs. Such differentiated support is consistent with the broader principle that the value of cognitive tools depends on the learner’s capacity to regulate their use.

### 4.5. Limitations and Future Research

Several limitations should be acknowledged. First, the cross-sectional survey does not establish temporal ordering or causal mediation. Although the model was theoretically directional, reverse and reciprocal explanations remain plausible. Students with lower perceived academic creativity may turn to GenAI more often or rely on it more strongly; dependence and perceived creativity may also reinforce one another over time. Longitudinal cross-lagged, experimental, and quasi-experimental studies are needed to separate these possibilities and test within-person change.

Second, all focal variables were self-reported in one sitting. The common-method diagnostics reduce some concerns but cannot remove shared-source bias. Future studies should include at least one behavioral anchor, such as prompt logs, AI interaction histories, draft revisions, source-verification records, divergent-thinking tasks, creative problem-solving tasks, or expert-rated academic products, and should separate predictor and outcome measurement across time or sources.

Third, perceived academic creativity was measured as creative self-perception in academic contexts, not as objective creative performance or general intelligence. Perceived and performance-based creativity may be related but are not interchangeable; self-reports capture students’ beliefs about their creative functioning and agency. Future work should test whether the present associations generalize to expert-rated products, divergent-thinking performance, reasoning, working memory, and other cognitive outcomes.

Fourth, GenAI use intensity was measured as a general construct. Students use GenAI for explanation, translation, brainstorming, summarization, writing assistance, coding, emotional support, and direct answer generation. These purposes may have different implications for dependence and creativity. Future studies should distinguish exploratory, reflective, and substitutive patterns of use and examine how each relates to cognitive development.

Fifth, the sample was limited to university students in China. Domestic tools such as DeepSeek, Kimi, Wenxin Yiyan, and Tongyi Qianwen were prominent in participants’ reported use, whereas the availability and institutional accessibility of some Western platforms can differ. These tool ecologies, together with assessment pressure, teacher expectations, and institutional policies, may shape use and dependence. University-level ICCs were at or below 0.003 in this sample, but only six universities were included. Future research should test the model across countries, institutional types, disciplines, platforms, and educational systems. Full measurement invariance across gender, discipline, and academic year was not tested; the unequal and in some cases small subgroup sizes made a comprehensive 45-item multi-group analysis outside the scope of the present study. Future research should examine configural, metric, and scalar invariance in samples designed for subgroup comparisons.

### 4.6. Implications for Theories of Intelligence and Cognitive Development

The findings also raise broader questions about how theories of intelligence and cognitive development should represent intellectual functioning when external intelligent systems are continuously available. Classical accounts were developed in settings in which reasoning, synthesis, evaluation, and problem solving were treated primarily as capacities of the individual (Spearman, 1904; Piaget, 1952). GenAI does not eliminate individual differences in cognitive ability, but it changes the conditions under which intellectual performance is produced. Observable performance may increasingly reflect the interaction among internal cognitive resources, the capabilities of the external system, and the learner’s capacity to regulate that interaction.

From a Spearmanian perspective, general intelligence may remain an important source of common variance across reasoning and learning tasks. However, AI-supported performance may no longer provide a direct indication of unaided cognitive ability. Students with similar internal resources may produce different outcomes because they differ in access, prompting strategies, verification, and integration of externally generated information. Conversely, similarly polished products may conceal substantial differences in independent reasoning. Contemporary theories and assessments may therefore need to distinguish unaided cognitive ability, AI-augmented performance, and the regulatory competence required to coordinate internal and external cognitive resources. This proposal extends rather than rejects the relevance of general intelligence by clarifying the performance conditions under which it is expressed.

Piagetian theory offers a complementary developmental interpretation. Cognitive development depends on active construction through assimilation, accommodation, and the resolution of disequilibrium (Piaget, 1952). GenAI may support these processes when it provides counterexamples, alternative explanations, or feedback that encourages learners to revise existing cognitive structures. Used in this way, the system can function as a scaffold for reconstruction. However, when GenAI supplies the problem representation, reasoning sequence, or conclusion before learners have engaged with uncertainty, it may reduce opportunities for self-generated exploration and accommodation. Immediate task completion can therefore improve without a corresponding change in the learner’s independent cognitive structures.

These considerations place AI literacy in a broader theoretical role. In addition to technical knowledge, AI literacy may represent a metacognitive and epistemic regulatory capacity: knowing when to seek external assistance, how to evaluate and transform it, and when to withhold assistance in order to preserve independent reasoning. The distinction between scaffolded and substitutive use thus concerns the boundary between internal and external cognition (Markauskaite et al., 2022; Risko & Gilbert, 2016). Intelligence assessment in AI-rich environments may accordingly need to evaluate output quality together with process ownership, source verification, transfer after tool withdrawal, and the quality of human–AI coordination.

The present study cannot establish these theoretical extensions. Its cross-sectional and self-report design does not show how sustained GenAI use changes general ability or developmental trajectories. The findings instead identify a pattern that motivates longitudinal, experimental, and performance-based research comparing unaided and AI-assisted performance, examining transfer when support is removed, and testing whether regulatory competence predicts durable learning. Such evidence will be necessary to determine how theories of intelligence and cognitive development should incorporate routinely available external intelligent tools.

## 5. Conclusions

The present study indicates that GenAI use intensity and perceived academic creativity are connected through opposing cross-sectional associations. Use intensity had a positive direct association with perceived academic creativity and a negative indirect association through cognitive dependence; the resulting net association was small. Higher AI literacy was associated with a weaker link between use intensity and dependence. These findings do not establish causal effects, but they identify a theoretically meaningful distinction between scaffolded and substitutive patterns of use.

The practical question is whether students remain intellectually responsible while using GenAI. Process-oriented assessment, critical AI literacy, source verification, and explicit cognitive boundaries may help educators distinguish support that extends students’ thinking from assistance that replaces it. More broadly, theories and assessments of intelligence may need to distinguish unaided ability, AI-augmented performance, and the regulatory competence that coordinates them. Longitudinal, behavioral, and experimental evidence is needed before drawing conclusions about changes in actual creative capacity.

## Figures and Tables

**Table 1:** Descriptive statistics and correlations among the study variables.

Variable	*M*	*SD*	1	2	3	4
1. GenAI Use Intensity	3.46	0.81	-			
2. AI Literacy	3.62	0.74	0.24 ***	-		
3. GenAI Cognitive Dependence	3.08	0.79	0.43 ***	−0.36 ***	-	
4. Perceived Academic Creativity	3.51	0.70	0.18 ***	0.52 ***	−0.47 ***	-

Note. *** *p* < .001.

**Table 2:** Reliability and convergent validity of the study variables.

Construct	Items	Std. Loadings	α	ω	CR	AVE	CITC
GenAI Use Intensity	6	0.687–0.846	0.887	0.891	0.889	0.573	0.621–0.783
AI Literacy	16	0.672–0.858	0.947	0.949	0.948	0.535	0.654–0.821
GenAI Cognitive Dependence	12	0.704–0.879	0.938	0.940	0.939	0.563	0.682–0.834
Perceived Academic Creativity	11	0.696–0.865	0.929	0.931	0.931	0.551	0.661–0.819

Note. CR = composite reliability; AVE = average variance extracted; CITC = corrected item-total correlation range.

**Table 3:** Comparison of the hypothesized measurement model with alternative models.

Model	*χ* ^2^	*df*	CFI	TLI	RMSEA	SRMR	ΔCFI	ΔRMSEA
Four-factor	2034.68	939	0.955	0.952	0.035	0.041	-	-
3-factor: GAIU + GCD	3568.24	942	0.892	0.886	0.055	0.071	−0.063	0.020
3-factor: AIL + PAC	4127.56	942	0.869	0.862	0.060	0.078	−0.086	0.025
Two-factor	6425.83	944	0.774	0.762	0.079	0.101	−0.181	0.044
One-factor	10,386.17	945	0.611	0.593	0.103	0.137	−0.344	0.068

Note. GAIU = GenAI use intensity; AIL = AI literacy; GCD = GenAI cognitive dependence; PAC = perceived academic creativity. Delta values are calculated relative to the hypothesized four-factor model. Because robust maximum likelihood estimation was used, scaled chi-square difference tests are preferable for formal nested comparisons; therefore, this table emphasizes comparative fit indices.

**Table 4:** Common method bias diagnostics.

Diagnostic Approach	Evidence	Interpretation
Procedural remedies	Anonymity, item separation, varied construct sections, and no right-or-wrong-answer instructions	Reduced evaluation apprehension and demand characteristics
Harman single-factor test	First unrotated factor = 31.84% of total variance	Below the commonly used 40% reference value
Single-factor CFA	*χ*^2^(945) = 10,386.17, *CFI* = 0.611, *TLI* = 0.593, *RMSEA* = 0.103, *SRMR* = 0.137	Poor fit; a single general factor did not represent the data well
Unmeasured latent method factor	Focal paths retained the same direction and statistical significance	Common method variance was unlikely to fully explain the main associations

Note. Common method bias cannot be ruled out completely because all focal variables were measured by self-report at one time point.

**Table 5:** Standardized path coefficients of the structural model.

Path	*β*	*SE*	95% *CI*	*p*
GAIU → GCD	0.36	0.04	[0.28, 0.44]	<.001
AIL → GCD	−0.40	0.03	[−0.46, −0.34]	<.001
GCD → PAC	−0.39	0.04	[−0.47, −0.31]	<.001
GAIU → PAC	0.18	0.04	[0.10, 0.26]	<.001
AIL → PAC	0.42	0.04	[0.34, 0.50]	<.001

Note. GAIU = GenAI use intensity; AIL = AI literacy; GCD = GenAI cognitive dependence; PAC = perceived academic creativity. Control variables included gender, academic year, disciplinary background, self-reported academic performance, length of GenAI use, and prior AI-related training. Weekly GenAI use frequency was excluded from the main model because of conceptual overlap with GAIU.

**Table 6:** Direct and indirect associations between GenAI use intensity and perceived academic creativity.

Effect	*β*	*SE*	95% *CI*	Interpretation
Direct effect: GAIU → PAC	0.180	0.040	[0.100, 0.260]	Positive association after accounting for GCD and AIL
Indirect effect: GAIU → GCD → PAC	−0.140	0.025	[−0.191, −0.094]	Negative indirect association through cognitive dependence

Note. GAIU = GenAI use intensity; GCD = GenAI cognitive dependence; AIL = AI literacy; PAC = perceived academic creativity. Based on the standardized direct and indirect estimates, the model-implied net association was small (β ≈ 0.040), indicating a competing-pathway pattern.

**Table 7:** Conditional indirect effects of GenAI use intensity on perceived academic creativity through cognitive dependence at different levels of AI literacy.

Level of AI Literacy	Effect of GAIU on GCD	Indirect Effect	*SE*	95% *CI*
Low AI literacy (−1 SD)	0.52 ***	−0.203	0.035	[−0.275, −0.139]
Mean AI literacy	0.34 ***	−0.133	0.027	[−0.188, −0.084]
High AI literacy (+1 SD)	0.16 **	−0.062	0.023	[−0.110, −0.022]
Index of moderated mediation	—	0.070	0.018	[0.037, 0.108]

Note. GAIU = GenAI use intensity; GCD = GenAI cognitive dependence. ** *p* < .01. *** *p* < .001. Conditional indirect effects were estimated from the latent moderated mediation model. Confidence intervals were obtained using Monte Carlo simulation for the latent moderated mediation model.

## Data Availability

The data that support the findings of this study are available from the corresponding author upon reasonable request. The data are not publicly available due to participant confidentiality and ethical restrictions.

## Supplementary Materials

The following supporting information can be downloaded at: https://www.mdpi.com/article/10.3390/jintelligence14080185/s1, Supplementary Materials: Scale Items and Domain Structure; Table S1: Provisional item wording and adaptation basis for GenAI use intensity, AI literacy, GenAI cognitive dependence, and perceived academic creativity; Table S2: Provisional domain structure and construct representation; Table S3: Pilot-sample item diagnostics (n = 96); Table S4: Reproducible split-sample CFA results; Table S5a: Complete Geomin-rotated four-factor EFA loading matrix (N = 936); Table S5b: Geomin factor correlations; Table S5c: HTMT matrix; Table S6: Weekly GenAI-use frequency sensitivity analysis; Table S7a: Nested LMS model-comparison diagnostics; Table S7b: LMS interaction and conditional effects reported in the main text; Table S8: University-level intraclass correlations.
