# Supplementary material for: Generative AI as an External Cognitive Tool: Cognitive Dependence, AI Literacy, and Perceived Academic Creativity
Source: J Intell. 2026 Aug 11;14(8):185. doi: 10.3390/jintelligence14080185 (PMC13514563; doi:10.3390/jintelligence14080185)
Supplement: Supplementary file 1 [file jintelligence-14-00185-s001.zip › jintelligence-4444645-supplementary.pdf]

Supplementary Appendix A

Scale Items and Domain Structure

Table S1. Provisional item wording and adaptation basis: GenAI use intensity

Code	Provisional domain/content	Proposed English item wording
GAIU1	Learning-content understanding	I use generative AI tools to help me understand difficult course content.
GAIU2	Assignment support	I use generative AI tools to help me complete assignments, reports, or course papers.
GAIU3	Information summarization	I use generative AI tools to summarize readings, lectures, or other learning materials.
GAIU4	Outline generation	I use generative AI tools to generate outlines or organize the initial structure of academic work.
GAIU5	Written-work revision	I use generative AI tools to revise or improve my written academic work.
GAIU6	Complex-task assistance	When a learning task is complex, I usually use generative AI to obtain assistance.

Note. Developed for the present study with reference to Chan and Hu (2023), Kim et al. (2025), and Strzelecki (2024). Proposed response scale: 1 = never to 5 = always. No reverse-keyed item is assumed in this draft.

Table S1. Provisional item wording (continued): AI literacy

Code	Provisional domain/content	Proposed English item wording
AIL1	Needs analysis	Before using generative AI, I can identify what learning problem I need to solve.
AIL2	Needs analysis	I can decide whether a learning task is suitable for generative AI assistance.
AIL3	Needs analysis	I can identify which parts of a learning task require my own thinking rather than AI assistance.
AIL4	Needs analysis	I can set a clear goal before asking generative AI for help.
AIL5	Prompt and language skills	I can write prompts that clearly describe the learning task I want generative AI to perform.
AIL6	Prompt and language skills	I can adjust my prompts to make generative AI responses better fit the learning task.
AIL7	Prompt and language skills	I can provide generative AI with relevant context, constraints, and examples when needed.
AIL8	Prompt and language skills	I can use follow-up questions to clarify or improve a generative AI response.
AIL9	Autonomous learning	I can use generative AI to support my learning without letting it replace my independent thinking.
AIL10	Autonomous learning	I can integrate useful generative AI output into my own learning plan or problem-solving process.
AIL11	Autonomous learning	I can decide when to stop using generative AI and continue the task independently.
AIL12	Autonomous learning	I can monitor whether using generative AI is actually helping me achieve my learning goal.
AIL13	Critical evaluation	I critically evaluate the accuracy of generative AI-generated content.
AIL14	Critical evaluation	I can identify possible limitations, biases, or fabricated information in generative AI responses.
AIL15	Critical evaluation	I verify important generative AI-generated information using reliable academic sources.
AIL16	Critical evaluation	I can judge whether using generative AI in a task is academically appropriate and responsible.

Note. Adapted from AI-literacy and GenAI-literacy frameworks (Jin et al., 2025; Laupichler et al., 2023; Long & Magerko, 2020; Ng et al., 2021). Proposed response scale: 1 = strongly disagree to 5 = strongly agree. No reverse-keyed item is assumed in this draft.

Table S1. Provisional item wording (continued): GenAI cognitive dependence

Code	Provisional domain/content	Proposed English item wording
GCD1	Cognitive outsourcing dependence	When I encounter a learning problem, my first reaction is usually to ask generative AI rather than think it through myself.
GCD2	Cognitive outsourcing dependence	I rely on generative AI to decide how to start difficult academic tasks.
GCD3	Cognitive outsourcing dependence	I often rely on generative AI to generate the basic ideas for assignments, papers, or reports.
GCD4	Cognitive outsourcing dependence	I depend on generative AI to organize or integrate information that I could otherwise process myself.
GCD5	Uncritical acceptance	I tend to accept generative AI responses without carefully checking the reasoning behind them.
GCD6	Uncritical acceptance	If a generative AI response sounds convincing, I usually use it without seeking additional evidence.
GCD7	Uncritical acceptance	I rarely question the conclusions suggested by generative AI during learning tasks.
GCD8	Uncritical acceptance	I allow generative AI outputs to shape my academic judgments even when I have not verified them.
GCD9	Weakened independent thinking	I find it difficult to develop ideas independently after becoming accustomed to generative AI assistance.
GCD10	Weakened independent thinking	I spend less time reasoning through academic problems myself because generative AI can provide an answer quickly.
GCD11	Weakened independent thinking	I feel less able to organize complex academic work without generative AI.
GCD12	Weakened independent thinking	Without generative AI, I feel less confident in completing complex learning tasks.

Note. Adapted from cognitive-offloading, technology-dependence, and AI-supported-learning research (Risko & Gilbert, 2016; Sparrow et al., 2011; Ward, 2021). Proposed response scale: 1 = strongly disagree to 5 = strongly agree. No reverse-keyed item is assumed in this draft.

Table S1. Provisional item wording (continued): Perceived academic creativity

Code	Provisional domain/content	Proposed English item wording
PAC1	Academic idea generation	I can propose original questions in course learning.
PAC2	Academic idea generation	I can generate several possible approaches to an academic problem.
PAC3	Academic idea generation	I can develop ideas that are both novel and useful for an assignment or project.
PAC4	Academic idea generation	I can suggest new explanations for issues discussed in class.
PAC5	Knowledge integration	I can connect knowledge from different courses or disciplines to form new ideas.
PAC6	Knowledge integration	I can reorganize existing information to produce a new academic perspective.
PAC7	Academic expression	I can propose creative ways to present or communicate academic content.
PAC8	Argument development	I can develop an original argument in academic writing.
PAC9	Critical problem finding	I can identify limitations in existing arguments, materials, or studies.
PAC10	Methodological creativity	I can propose possible improvements to an existing method, solution, or research design.
PAC11	Creative self-perception	I consider myself capable of producing creative work in academic contexts.

Note. Adapted from the scholarly-creativity domain of the Kaufman Domains of Creativity Scale (Kaufman, 2012) and contextualized for higher education. Proposed response scale: 1 = strongly disagree to 5 = strongly agree. No reverse-keyed item is assumed in this draft.

Table S2. Provisional domain structure and construct representation

Construct	Content domain	Provisional item range	Interpretive content	Main-model representation
GenAI use intensity (GAIU)	Task breadth / use contexts	GAIU1-GAIU6	Learning-content understanding, assignment support, summarization, outline generation, written-work revision, and complex-task assistance	One first-order latent construct with six indicators
AI literacy (AIL)	Needs analysis	AIL1-AIL4	Identify the learning need, task suitability, learner-controlled components, and the goal for AI assistance	Overall AIL latent construct; 16 item indicators in the main SEM
AI literacy (AIL)	Prompt and language skills	AIL5-AIL8	Formulate, refine, contextualize, and follow up prompts	Overall AIL latent construct; 16 item indicators in the main SEM
AI literacy (AIL)	Autonomous learning	AIL9-AIL12	Integrate AI support while retaining independent control and monitoring learning progress	Overall AIL latent construct; 16 item indicators in the main SEM
AI literacy (AIL)	Critical evaluation	AIL13-AIL16	Evaluate accuracy, limitations, evidence, and academic appropriateness	Overall AIL latent construct; 16 item indicators in the main SEM
GenAI cognitive dependence (GCD)	Cognitive outsourcing dependence	GCD1-GCD4	Outsource task initiation, idea generation, information organization, and integration	Overall GCD latent construct; 12 item indicators in the main SEM
GenAI cognitive dependence (GCD)	Uncritical acceptance of AI outputs	GCD5-GCD8	Accept convincing outputs with limited questioning, evidence checking, or judgment verification	Overall GCD latent construct; 12 item indicators in the main SEM
GenAI cognitive dependence (GCD)	Weakened independent thinking	GCD9-GCD12	Reduced independent ideation, reasoning, organization, and confidence without AI assistance	Overall GCD latent construct; 12 item indicators in the main SEM
Perceived academic creativity (PAC)	Scholarly / academic creativity	PAC1-PAC11	Self-perceived idea generation, knowledge integration, academic expression, argument development, problem finding, and methodological creativity	One first-order latent construct with 11 indicators

Pilot and Measurement Evidence

Table S3. Pilot-sample item diagnostics (n = 96)

Construct	Items	α	Corrected item-total r	Item mean range	Missing
GenAI use intensity	6	.852	.587-.733	3.34-3.54	0
AI literacy	16	.901	.489-.695	3.35-3.73	0
GenAI cognitive dependence	12	.937	.684-.738	3.08-3.34	0
Perceived academic creativity	11	.904	.510-.727	3.22-3.46	0

Note. Pilot cases were independent of the formal sample. α is Cronbach's alpha.

Table S4. Reproducible split-sample CFA results

Sample	n	Estimator	χ^2	df	p	CFI	Raw TLI	Reported TLI	RMSEA [90% CI]	SRMR
Calibration	468	MLR	695.573	939	1.000	1.000	1.021	1.000	0.000 [0.000, 0.000]	0.024
Validation	468	MLR	695.990	939	1.000	1.000	1.021	1.000	0.000 [0.000, 0.000]	0.024

Note. Seed = 20250801; Python random.Random (MT19937). TLI values above 1.00 were bounded at 1.000 for presentation; raw values are retained. Both models terminated normally without warnings or errors.

Complete Cross-Loadings and Discriminant Validity

Table S5a. Complete Geomin-rotated four-factor EFA loading matrix (N = 936)

Item	Target	Factor 1 GAIU	Factor 2 AIL	Factor 3 GCD	Factor 4 PAC	Max |cross|
GAIU1	GAIU	**.622**	.024	.032	.000	.032
GAIU2	GAIU	**.712**	-.005	.023	.037	.037
GAIU3	GAIU	**.768**	-.005	-.018	-.014	.018
GAIU4	GAIU	**.761**	.018	.033	.019	.033
GAIU5	GAIU	**.815**	-.013	-.020	-.008	.020

Table S5a. Complete EFA loading matrix (continued)

Item	Target	Factor 1 GAIU	Factor 2 AIL	Factor 3 GCD	Factor 4 PAC	Max |cross|
GAIU6	GAIU	**.799**	.014	.016	-.002	.016
AIL1	AIL	.019	**.626**	-.013	.031	.031
AIL2	AIL	-.016	**.655**	.014	.007	.016
AIL3	AIL	-.007	**.650**	.010	.019	.019
AIL4	AIL	.012	**.662**	-.005	.008	.012
AIL5	AIL	.026	**.677**	-.023	-.019	.026
AIL6	AIL	.010	**.701**	.001	-.002	.010
AIL7	AIL	.006	**.702**	-.018	-.009	.018
AIL8	AIL	.020	**.697**	-.033	.003	.033
AIL9	AIL	.000	**.720**	-.007	.017	.017
AIL10	AIL	-.017	**.744**	.015	.018	.018
AIL11	AIL	.021	**.746**	-.010	-.016	.021
AIL12	AIL	-.012	**.768**	-.003	.012	.012
AIL13	AIL	.008	**.754**	-.011	.016	.016
AIL14	AIL	-.005	**.788**	.011	.014	.014
AIL15	AIL	.010	**.799**	-.015	.002	.015
AIL16	AIL	-.005	**.812**	-.005	.022	.022
GCD1	GCD	-.005	.000	**.663**	-.010	.010
GCD2	GCD	.030	-.032	**.650**	-.021	.032
GCD3	GCD	.041	-.018	**.633**	-.035	.041
GCD4	GCD	.040	-.002	**.660**	-.034	.040
GCD5	GCD	-.013	.007	**.734**	-.001	.013
GCD6	GCD	.014	-.017	**.722**	-.005	.017
GCD7	GCD	-.013	-.002	**.777**	.013	.013
GCD8	GCD	.010	-.013	**.744**	-.021	.021
GCD9	GCD	-.030	-.007	**.811**	.024	.030
GCD10	GCD	.008	.004	**.807**	.000	.008
GCD11	GCD	.013	-.020	**.816**	.003	.020
GCD12	GCD	.020	.024	**.819**	-.025	.025
PAC1	PAC	.009	.019	-.042	**.621**	.042
PAC2	PAC	-.008	.013	.006	**.665**	.013
PAC3	PAC	.023	.008	-.038	**.660**	.038

Table S5a. Complete EFA loading matrix (continued)

Item	Target	Factor 1 GAIU	Factor 2 AIL	Factor 3 GCD	Factor 4 PAC	Max |cross|
PAC4	PAC	-.032	.026	.008	**.697**	.032
PAC5	PAC	.028	-.003	-.019	**.715**	.028
PAC6	PAC	.015	-.024	-.019	**.746**	.024
PAC7	PAC	-.018	.031	.032	**.758**	.032
PAC8	PAC	-.010	.002	-.007	**.773**	.010
PAC9	PAC	.046	.022	-.042	**.737**	.046
PAC10	PAC	.004	-.024	-.002	**.808**	.024
PAC11	PAC	-.009	.021	.016	**.814**	.021

Note. Extraction = MLR; rotation = Geomin oblique; row standardization = correlation. Target loadings are bold. Factor labels were assigned from the intended item blocks; all loadings are shown. The maximum absolute cross-loading was .046 (PAC9 on Factor 1).

Table S5b. Geomin factor correlations

Factor	1	2	3	4
1. GAIU	1.000			
2. AIL	.256	1.000		
3. GCD	.466	-.369	1.000	
4. PAC	.196	.534	-.488	1.000

Table S5c. HTMT matrix

Construct	1	2	3	4
1. GAIU	1.000			
2. AIL	.262	1.000		
3. GCD	.471	.382	1.000	
4. PAC	.198	.554	.503	1.000

Note. HTMT was computed as the mean absolute heterotrait correlation divided by the geometric mean of the two mean absolute monotrait correlations. Range = .198-.554; GAIU-GCD = .471.

Robustness and Model Comparison

Table S6. Weekly GenAI-use frequency sensitivity analysis

Path	Without weekly β	HC3 SE	With weekly β	HC3 SE	p (with weekly)
GCD on GAIU	.539	.030	.534	.039	< .001
GCD on AIL	-.467	.029	-.468	.029	< .001
GCD on GAIU × AIL	-.146	.021	-.146	.021	< .001
PAC on GCD	-.519	.032	-.519	.032	< .001
PAC on GAIU	.347	.033	.346	.041	< .001
PAC on AIL	.211	.035	.211	.035	< .001
Weekly frequency on GCD	--	--	.008	.036	0.826
Weekly frequency on PAC	--	--	.002	.035	0.961

Note. Observed-score sensitivity models used standardized scale means, identical demographic/learning controls, and HC3 standard errors. The focal coefficients were substantively unchanged after weekly frequency was added.

Table S7a. Nested LMS model-comparison diagnostics

Model	Free parameters	H0 loglikelihood	MLR scaling	AIC	BIC	Adjusted BIC
Restricted: INT -> GCD fixed 0	169	-46419.648	.9503	93177.296	93995.529	93458.799
Free: INT -> GCD estimated	170	-46401.249	.9485	93142.497	93965.572	93425.666

Note. MLR-scaled likelihood-ratio test: $\Delta\chi^2(1) = 57.113$, $p < .001$; difference-test scaling correction cd = .6443. These statistics are relative-fit diagnostics for the nested interaction comparison. R^2 values from the separate nested run are omitted so that supplementary reporting remains on the same effect-estimate basis as the main text.

Table S7b. LMS interaction and conditional effects reported in the main text

Effect	β	SE	95% CI	p
Interaction: GAIU × AIL -> GCD	-.180	.040	--	< .001
Simple slope at low AIL (-1 SD)	.520	.050	--	< .001
Simple slope at mean AIL	.340	.040	--	< .001
Simple slope at high AIL (+1 SD)	.160	.050	--	.002
Conditional indirect effect: low AIL	-.203	.035	[-.275, -.139]	--
Conditional indirect effect: mean AIL	-.133	.027	[-.188, -.084]	--
Conditional indirect effect: high AIL	-.062	.023	[-.110, -.022]	--
Index of moderated mediation	.070	.018	[.037, .108]	--

Note. Values reproduce the standardized estimates in manuscript Table 7 and its accompanying text. From low to high AI literacy, the GAIU-to-GCD slope decreased by .36, or approximately 69%; the absolute conditional indirect association decreased from .203 to .062, or approximately 69%.

Table S8. University-level intraclass correlations

Construct	Universities	n per university	ICC(1), bounded	Raw moment estimate
GenAI use intensity	6	156	.003	.003
AI literacy	6	156	.000	.000
GenAI cognitive dependence	6	156	.000	-.006
Perceived academic creativity	6	156	.000	.000

Note. The negative raw moment estimate for GCD reflects sampling fluctuation around zero and was bounded at zero for variance interpretation. All ICCs were at or below .003.
